# Multi-functional reactively-sputtered copper oxide electrodes for supercapacitor and electro-catalyst in direct methanol fuel cell applications

**DOI:** 10.1038/srep21310

**Published:** 2016-02-18

**Authors:** Sambhaji M. Pawar, Jongmin Kim, Akbar I. Inamdar, Hyeonseok Woo, Yongcheol Jo, Bharati S. Pawar, Sangeun Cho, Hyungsang Kim, Hyunsik Im

**Affiliations:** 1 Division of Physics and Semiconductor Science, Dongguk University, Seoul 04620, Korea

## Abstract

This work reports on the concurrent electrochemical energy storage and conversion characteristics of granular copper oxide electrode films prepared using reactive radio-frequency magnetron sputtering at room temperature under different oxygen environments. The obtained films are characterized in terms of their structural, morphological, and compositional properties. X-ray diffraction, X-ray photoelectron spectroscopy and scanning electron microscope studies reveal that granular, single-phase Cu_2_O and CuO can be obtained by controlling the oxygen flow rate. The electrochemical energy storage properties of the films are investigated by carrying out cyclic voltammetry, galvanostatic charge/discharge and electrochemical impedance spectroscopy tests. The electrochemical analysis reveals that the Cu_2_O and CuO electrodes have high specific capacitances of 215 and 272 F/g in 6 M KOH solution with a capacity retention of about 80% and 85% after 3000 cycles, respectively. Cyclic voltammetry and chronoamperometry are used to study the electrochemical energy conversion properties of the films via methanol electro-oxidation. The results show that the Cu_2_O and CuO electrodes are electro-catalytically active and highly stable.

The rapid depletion of fossil fuels and the ever-increasing concern over environmental pollution has motivated intensive research and development of novel materials to improve the performance of advanced energy devices^1^,^2^. Recently, there has been an increased interest in supercapacitors because they possess a higher energy density and power density than batteries, fast charging/discharging rates, and a long cycle life^3^,^4^,^5^. Direct methanol fuel cells (DMFCs) have also attracted a considerable amount of interest due to the abundance of their raw materials, high power density, fast recharge, and low operating temperatures^2^,^6^. Nevertheless, it will be useful to prepare cost-effective, multi-functional electrode materials with reasonably good performance capabilities in order to further develop advanced supercapacitors and DMFCs.

Among various factors, a relatively high-cost, complex-fabrication and the low-activity of electrode materials are the main obstacles for using supercapacitors and DMFCs in large-scale applications^6^,^7^. Therefore, many efforts have been taken to search for alternative inexpensive novel electrode materials^8^,^9^,^10^,^11^ that can be fabricated at low cost and can achieve high levels of activity. Of the different transition metal oxides that are available, copper oxides are a promising material due to their low cost, larger abundance, chemical stability and environmentally friendly nature. Copper oxides have a desirable theoretical capacitance value of about 1800 F/g^12^, and various nanostructured copper oxides have been synthesized using a variety of methods^12^,^13^,^14^,^15^,^16^,^17^,^18^,^19^. The CuO nanowires deposited using anodization and electro-spun techniques show specific capacitances of 212 and 620 F/g^12^,^20^, respectively, whereas chemically synthesized Cu(OH)_2_/CuO nano-sheets exhibit maximum specific capacitances of 569 and 790 F/g^17^,^21^. However, the electrochemical properties of the chemically deposited materials are strongly dependent on their microstructure (morphology and dimension) and oxidation states, which are very difficult to reliably control during chemical synthesis^22^.

To this end, reactive magnetron sputtering offers a simple, one-step, and relatively cost-effective technique that can be used for large-scale applications. It can achieve high deposition rates, uniform deposition over large-area substrates, and allows for easy control over the composition, oxidation states and phases of the deposited materials. However, to the best of our knowledge, reactively-sputtered copper oxide films prepared at room temperature have been rarely studied for use in supercapacitors and as electro-catalysts in the electro-oxidation of methanol.

In this work, we synthesized granular copper oxide (Cu_2_O and CuO) films at room temperature via reactive RF magnetron sputtering under different oxygen environments, and we studied their physical and electro-chemical properties for their possible use as multi-functional electrodes in advanced energy devices. We evaluated their electro-chemical supercapacitive properties and electro-catalytic performance for methanol electro-oxidation in an alkaline medium.

## Results and Discussion

**Figure 1:**
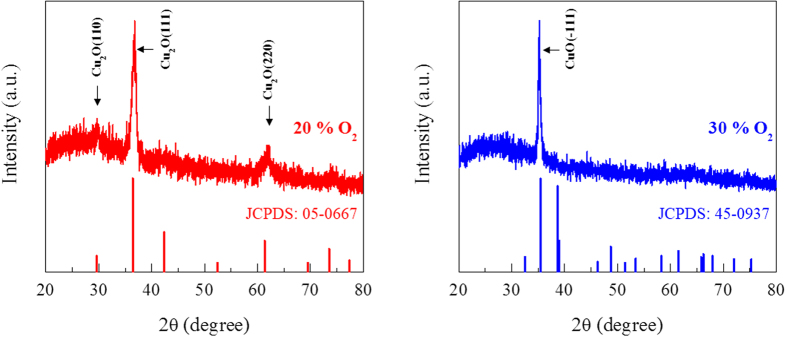
XRD patterns for the standard values of JCPDS [Nos. 05–0667 and 45–0937] and granular copper oxide films obtained by sputtering Cu at room temperature with different oxygen flow rates of 20% and 30%. Afterwards, the copper oxides prepared at the 20% and 30% oxygen flow rates are referred to as Cu_2_O and CuO, respectively. The accurate chemical composition of the films is summarized in Supplementary Table 1.

Figure 1 shows the X-ray diffraction patterns of the copper oxide electrode films deposited with 20% and 30% oxygen flow rates during reactive magnetron sputtering of a pure copper target. The film deposited at a 10% oxygen flow rate had shown strong (111) and (200) diffraction peaks for Cu and weak (111) diffraction peak for Cu_2_O (See Supplementary Information Fig. S1). As the oxygen flow rate increases from 10% to 20%, the deposited film is then converted into single-phase cubic Cu_2_O with (110), (111) and (220) diffraction peaks [JCPDS file No. 05-0667]. The films deposited at a 30% oxygen flow rates exhibit a crystalline structure in which Cu_2_O is transformed into single-phase monoclinic CuO with a (−111) orientation [JCPDS file No. 45–0937]. As the oxygen flow rate increases further above 30%, the monoclinic CuO (−111) phase remains unchanged (See Supplementary Information, Fig. S1). The XRD results confirm that the crystalline structure of the film is strongly dependent on the oxygen flow rate. Afterwards, copper oxide films prepared at the 20% and 30% oxygen flow rates will be referred to as Cu_2_O and CuO, respectively.

**Figure 2:**
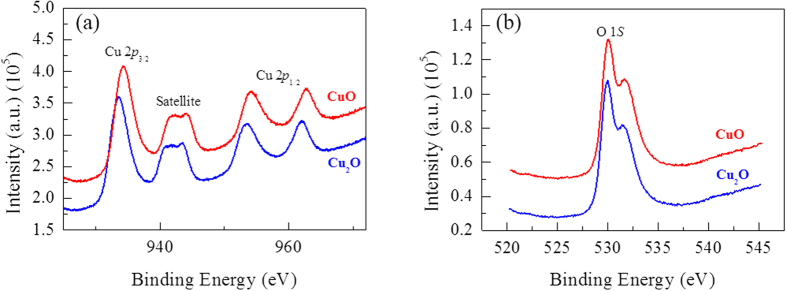
Core level XPS measurements of the Cu2O and CuO granular films. (**a**) Core level Cu 2p spectra. (**b**) Core level O 1s spectra.

Figure 2(a,b) shows the XPS spectra for the Cu 2p core level and O 1s core level of the copper oxide films. The main peaks and the shake-up peaks for both Cu 2p_3/2_ and Cu 2p_1/2_ are observed, and the shake-up peaks appear with a binding energy of 10 eV higher than that of the main Cu 2p_3/2_ and Cu 2p_1/2_ peaks. The Cu 2p_3/2_ peak is observed to have systematically shifted towards a higher binding energy from 933.54 to 934.26 eV as the oxygen flow rates increased from 20% to 30%. This indicates that the oxidation state of Cu changed from the 1 + oxidation state to the 2 + oxidation state. The shift in the binding energy is in good agreement with the Cu 2p_3/2_ peak values reported for both Cu_2_O and CuO^23^,^24^. The binding energy values for the O 1s peak in Cu_2_O are slightly higher than those for CuO by ~0.6 eV. This change is too small to be detected in our deposited samples, so all peak positions appear at nearly the same binding energy values of ~530 eV. The XRD and XPS analyses reveal that the copper oxide electrodes with two different phases (Cu_2_O and CuO) can be obtained by controlling the oxygen flow rate (See Supplementary Information, Table 1).

**Figure 3:**
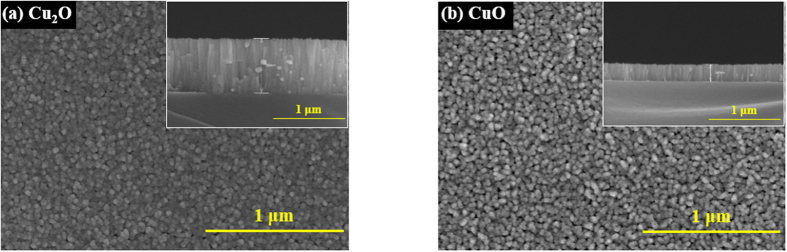
Film Morphology. Plane and cross-sectional (inset) SEM images of (**a**) Cu_2_O and (**b**) CuO electrode films. The CuO film has larger porosity than the Cu_2_O film.

Figure 3 shows plane and cross-sectional (inset) scanning electron microscope images of the Cu_2_O and CuO electrode films. The film deposited at a 10% oxygen flow rate has a compact morphology with a film thickness of about 760 nm (See Supplementary Information, Fig. S2). As the oxygen flow rate increases from 20% to 40%, the deposited films exhibit a granular, porous morphology, but the thicknesses of the films decreases below 300 nm. This decrease in the film thickness is due to the reduction in the sputtering rate at a higher oxygen flow rate^15^.

**Figure 4:**
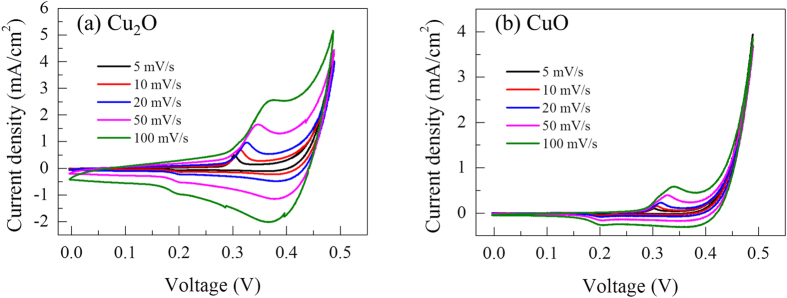
Cyclic voltammograms (CVs) recorded at different scan rates; 5, 10, 20, 50, and 100 mV/s for (a) Cu_2_O and (b) CuO, in a 6 M KOH electrolyte.

Cyclic voltammetry (CV) was employed to determine the supercapacitive properties of the copper oxide electrodes. Figure 4 shows cyclic voltammograms (CVs) at different scan rates in the potential range from 0 to 0.5 V/SCE in a 6 M KOH electrolyte. The selection of 6 M KOH is because a higher specific capacitance can be obtained at higher KOH concentration with upper limit of 6 M^12^,^17^,^20^. The anodic and cathodic peaks appear in the CV curve that corresponds to the Cu_2_O/CuO redox reaction, according to the following electrochemical reaction^12^:


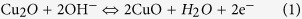



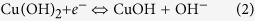


**Figure 5:**
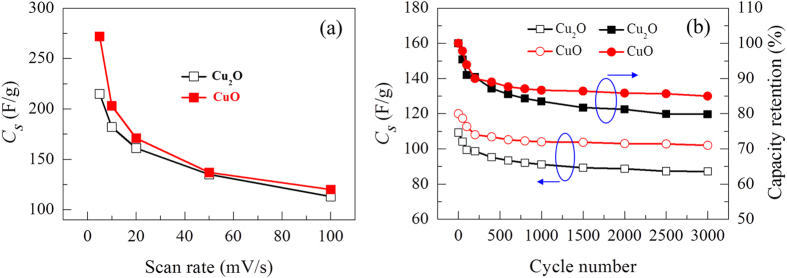
Specific capacitance (C_S_) calculated from the CV measurements. (**a**) *C*_*S*_ as a function of scan rate. (**b**) *C*_*S*_ and capacity retention (%) as a function of cycle number of the copper oxide electrode films at a scan rate of 100 mV/s.

The current under the curve gradually increases as the scan rate increases, which indicates that the voltammetric current is directly proportional to the scan rate used in the CV measurements, as expected for an ideally capacitive behavior^18^. In addition, as the scan rate increases, the anodic peak shifts towards the positive potential, while the cathodic peak shifts towards the negative potential because of the increased internal resistance of the electrode^25^. The specific capacitance (*C*_*S*_) of the deposited copper oxide electrodes can be calculated by using the following equation^25^,^26^


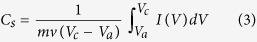


where *v* is the potential scan rate (mV/s), (*V*_*c*_ − *V*_*a*_) is the potential range, *I* denotes the response current, and *m* is the weight of the electrode film. Figure 5(a) shows the specific capacitance of the Cu_2_O and CuO electrodes measured at different scan rates. It is observed that the specific capacitance decreases as the scan rate increases. The decrease in the specific capacitance is attributed to the presence of inner active sites that cannot completely sustain redox transitions at higher scan rates^25^,^26^. Hence, the specific capacitance obtained at a slow scan rate could be considered as the full utilization of the electrode material. The maximum specific capacitances of the Cu_2_O and CuO electrodes are found to be 215 and 272 F/g, respectively. Figure 5(b) shows the specific capacitance at a scan rate of 100 m V/s and capacity retention as a function of the cycle number for up to 3000 cycles. The specific capacitance loss for both the electrodes occurs within the first 200 cycles. This may be due to degradation of the electrode materials^16^. After that, the capacitance stabilizes because the direct growth of copper oxide on the collector enable good mechanical adhesion and fast charge transfer without peeling off the active material during cycling^27^. Hence, the Cu_2_O and CuO electrodes retain 80% and 85% of their initial specific capacitance after 3000 cycles, demonstrating good cycling stability.

**Figure 6:**
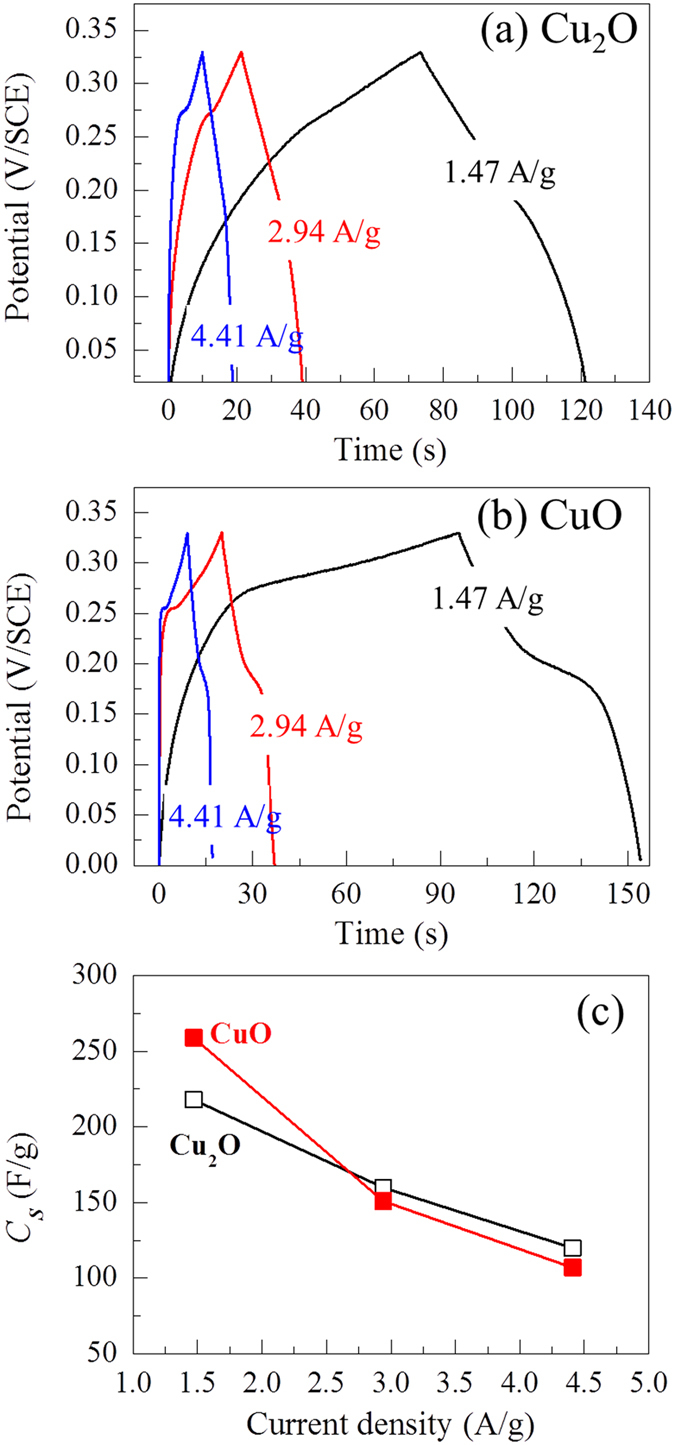
Galvanostatic charge-discharge (GCD) measurements at different current densities. (**a**) GCD curves for Cu_2_O and (**b**) GCD curves for CuO, within the potential window of 0 and 0.33 V (vs. SCE). (**c**) *C*_*S*_ calculated from the GCD measurements at different current densities.

The galvanostatic charge-discharge (GCD) measurements of the electrodes were measured using chronopotentiometry form 0 to 0.33 V at different current densities (1.47 – 4.41 A/g), as shown in Fig. 6. The shape of the measured charging and discharging curves is not an ideal straight line, suggesting that the observed capacitive behavior is a result of the Faradic redox reaction^12^. The charging process of both electrodes occurs above 0.1 V in the KOH electrolyte because of the oxidation of the copper oxide electrode and, in the discharging process, no potential drop is observed. This indicates a good interfacial contact between the active material and the substrate during the charge/discharge process^28^,^29^. From the GCD measurements, the specific capacitance (*C*_*S*_) of the electrodes can be calculated using the following equation:


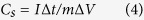


where *I*, Δ*t* and *m* are the response current, discharge time and weight of the electrode film. Δ*V* is the potential change during discharge. Figure 6(c) shows the specific capacitance as a function of the current density. The decrease in the specific capacitance with higher current densities is due to the redox reaction that takes place at the electrolyte/electrode interface^30^. These *C*_*S*_ values for different current densities are in good agreement with the results that were obtained from the cyclic voltammetry tests in Fig. 4.

**Figure 7:**
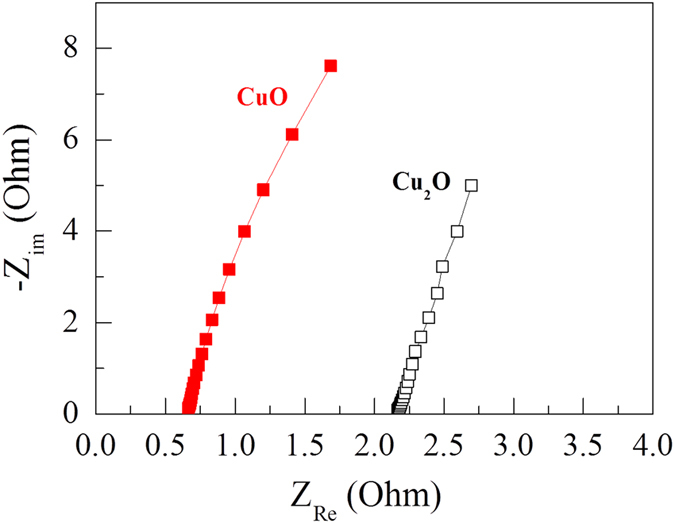
Electrochemical impedance spectra for the copper oxide electrodes in the frequency range from 1 MHz to 1 Hz.

In order to investigate the charge transfer at the electrode/electrolyte interface, electrochemical impedance spectroscopy (EIS) measurements were carried out before cycling in the frequency range from 1 MHz to 1 Hz. Figure 7 shows the impedance spectra for the Cu_2_O and CuO electrodes. The spectra present inclined lines over the entire frequency region, which is characteristic of supercapacitive behavior^28^. As the oxygen content in the film increases from Cu_2_O to CuO, the equivalent series resistance at the electrolyte/electrode interface decreases considerably from 2.1 Ω to 0.64 Ω, respectively. The lower resistance for the CuO electrode is presumably a result of the more conductive and porous structure of the material, which has a larger electroactive surface area and thus provides more active sites for Faradaic reactions and electrolyte permeation^31^,^32^. This suggests that the CuO electrode is more suitable for use in supercapacitor applications.

**Figure 8:**
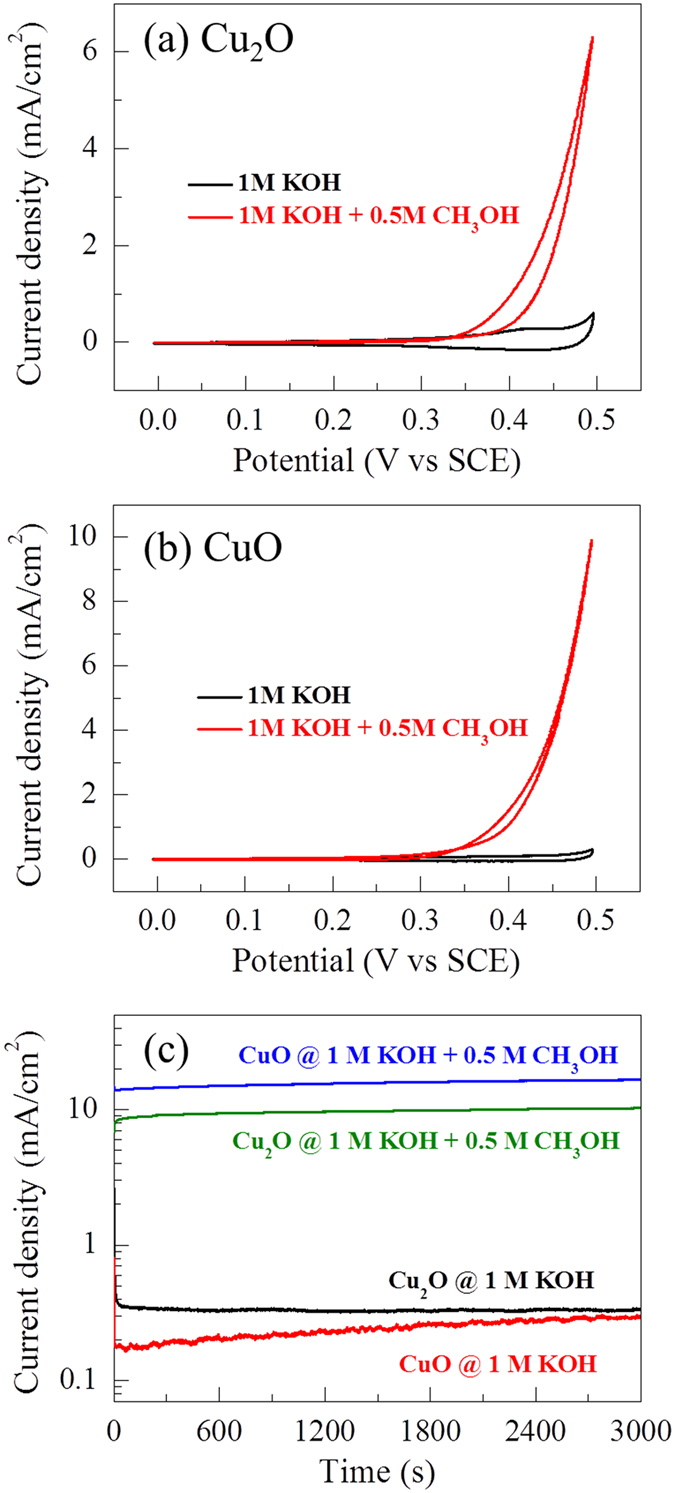
Electrocatalytic activity. Cyclic voltammograms (CVs) at a scan rate of 10 mV/s for (**a**) Cu_2_O and (**b**) CuO. (**c**) Chronoamperometry (CA) curves at a potential of 0.5 V (vs. SCE) for Cu_2_O and CuO electrodes measured in 1 M KOH and (1M KOH + 0.5 M CH_3_OH) electrolytes.

So far, we have presented the electrochemical energy storage properties of the Cu_2_O and CuO electrodes. To demonstrate their use in another energy device application, we conducted CV and chronoamperometry measurements to investigate their electrochemical energy conversion performance in terms of their properties as electro-catalysts for methanol electro-oxidation. Here, we have used 0.5 M Methanol to form the maximum possible quantity of Cu(OH)_2_ and to avoid an unwanted chemical reaction at a higher concentration of methanol, which reduces the quantity of Cu(OH)_2_ 10,33,34. Figure 8(a,b) shows the CV curves measured at a scan rate of 10 mV/s for the Cu_2_O and CuO electrodes in 1 M KOH and (1M KOH + 0.5 M CH_3_OH) electrolytes. In a 1 M KOH electrolyte, a pair of broad redox peaks are observed that originate mainly from the charge transfer process of the solid-state redox couple. After the addition of 0.5 M methanol in to the 1 M KOH electrolyte, during the forward scan, the anodic current density remains nearly the same up to 0.3 V and, above this potential, the copper oxide electrode surface is converted into CuOOH in the KOH electrolyte. Also, methanol is oxidized at a certain potential, which forms Cu(OH)_2_ and CO_2_ with a sharp increase in the anodic current density. This indicates that the electro-oxidation of methanol takes place on the surface of the electrodes^10^,^18^,^33^,^34^.

The corresponding electro-catalytic mechanism for methanol electro-oxidation can be expressed as


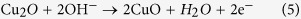



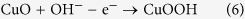



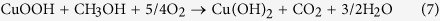


It is well known that the onset potential and the anodic current density are two important parameters for methanol electro-oxidation. The results in Fig. 8(a,b) show that the onset potential for both electrodes is the same (~0.42 V). However, the anodic current density of the CuO electrode is much higher than that of the Cu_2_O electrode. This indicates that the CuO electrode is more electro-catalytically active as compared to the Cu_2_O electrode. The electrochemical stability of the Cu_2_O and CuO electrodes is tested using chronoamperometry at 0.5 V (vs. SCE) for 3000 seconds, and the results are shown in Fig. 8(c). Without addition of methanol in the KOH electrolyte, the current densities of both the chronoamperometry curves are observed to be less than 0.5 μA/cm^2^. However, after the addition of 0.5 M methanol, the current densities for the Cu_2_O and CuO electrodes are about 10 and 16 mA/cm^2^ in the steady-state region and do not show any decay up to 3000 seconds. The results of the electro-catalytic performance clearly indicate that the potential of the reactively-sputtered copper oxide electrodes has met the essential requirements for both a high electro-catalytic activity and long-term stability for use as anodic materials in DMFCs.

## Conclusions

In conclusion, we have successfully synthesized copper oxide (Cu_2_O and CuO) electrode films at room temperature via reactive sputtering, and these can be used as multi-functional electrodes in various applications. The structural and morphological studies reveal that single-phase cubic Cu_2_O or monoclinic CuO can be obtained by controlling the oxygen flow rate. The CuO electrode film exhibits a maximum specific capacitance of 272 F/g at a scan rate of 5 mV/s in a 6 M KOH electrolyte, with a capacitive retention of about 85% after 3000 cycles. The results of the electro-catalytic study indicate that the reactively-sputtered copper oxide films are also suitable for use in methanol oxidation. Specifically, the CuO electrode shows a high electro-catalytic activity and an excellent long-term stability.

## Method

### Synthesis of copper oxide (Cu_2_O and CuO) electrode films

Copper oxide electrode films were deposited on 1 × 1 cm^2^ stainless steel substrates by using reactive radio frequency (RF) magnetron sputtering at room temperature under various oxygen environments. The stainless steel substrates were rinsed with acetone, methanol and deionized water in an ultrasonic bath and were then dried with nitrogen gas. The copper oxide films were also prepared for special purposes on different substrates, such as glass and silicon, and the substrates were fixed on a rotating substrate holder with a rotational speed of 10 rpm. Before film deposition, the chamber was evacuated to a base pressure of 3 × 10^**−**6^ Torr.

A pure copper target (Cu) was used for the film deposition, and the RF power for the Cu target was fixed to 100 W with a working pressure of 10 mTorr. Prior to film deposition, the target was pre-sputtered for 10 min to remove any possible contaminants. The total Ar and O_2_ flow rate remained constant at 30 sccm, and the O_2_ flow varied from 10% to 40% of the total flow rate. The copper oxide electrode films were then deposited for 15 min. The weight of copper oxide films deposited on the stainless steel substrates was determined using a sensitive microbalance and calculated by the weight difference. The weight of the copper oxide electrode films deposited at 10%, 20%, 30% and 40% are 2.1, 1.6, 0.34 and 0.30 mg, respectively.

### Material characterization

The structural properties of the deposited films were measured via high-resolution X-ray diffraction (XRD, X’pert PRO, Philips, Eindhoven, Netherlands) operating at 40 kV and 30 mA. The surface morphology and the composition of the films was determined using field emission scanning electron microscopy (FE-SEM, Model: JSM-6701F, Japan). X-ray photoelectron spectroscopy (XPS, Physical Electronics PHI 5400, USA) with a monochromatic MgKα (1253.6 eV) radiation source was used to characterize the chemical state of the deposited films.

### Measurement of the electrochemical supercapacitor performance

The electrochemical properties of the electrode films were measured by conducting cyclic voltammetry (CV), galvanostatic charge/discharge (GCD) and electrochemical impedance spectroscopy (EIS) tests using a potentiostat (Princeton Applied Research, VersaSTAT 3) with a specially-designed three electrode system with a solution of 6 M KOH as the electrolyte. A Pt foil was used as the counter electrode, a saturated calomel electrode (SCE) as the reference electrode, and the deposited films as the working electrode.

### Measurement of the electro-catalytic performance

Cyclic voltammetry (CV) and chronoamperometry (CA) were carried out at room temperature to measure the electro-catalytic properties of the copper oxide electrodes. The electro-catalytic activity of the electrodes for methanol oxidation was investigated using a potentiostat (Princeton Applied Research, VersaSTAT 3) in a standard three-electrode system. Copper oxide was used as a working electrode, a Pt foil as the counter electrode and SCE as the reference electrode. Solutions containing 1 M KOH without and with 0.5 M Methanol were used as the electrolyte.

## Additional Information

**How to cite this article**: Pawar, S. M. *et al*. Multi-functional reactively-sputtered copper oxide electrodes for supercapacitor and electro-catalyst in direct methanol fuel cell applications. *Sci. Rep.* **6**, 21310; doi: 10.1038/srep21310 (2016).

## Supplementary Material

Supplementary Information
