# Supplementary material for: Multi-functional reactively-sputtered copper oxide electrodes for supercapacitor and electro-catalyst in direct methanol fuel cell applications
Source: Sci Rep. 2016 Feb 18;6:21310. doi: 10.1038/srep21310 (PMC4758041; doi:10.1038/srep21310)
Supplement: Supplementary Information [file srep21310-s1.pdf]

## Supporting Information

# MULTI-FUNCTIONAL REACTIVELY-SPUTTERED COPPER OXIDE ELECTRODES FOR SUPERCAPACITOR AND ELECTRO-CATALYST IN DIRECT METHANOL FUEL CELL APPLICATIONS

Sambhaji M. Pawar, Jongmin Kim, Akbar I. Inamdar, Hyeonseok Woo, Yongcheol Jo, Bharati S. Pawar, Sangeun Cho, Hyungsang Kim, Hyunsik Im

Division of Physics and Semiconductor Science, Dongguk University, Seoul 04620, Korea.

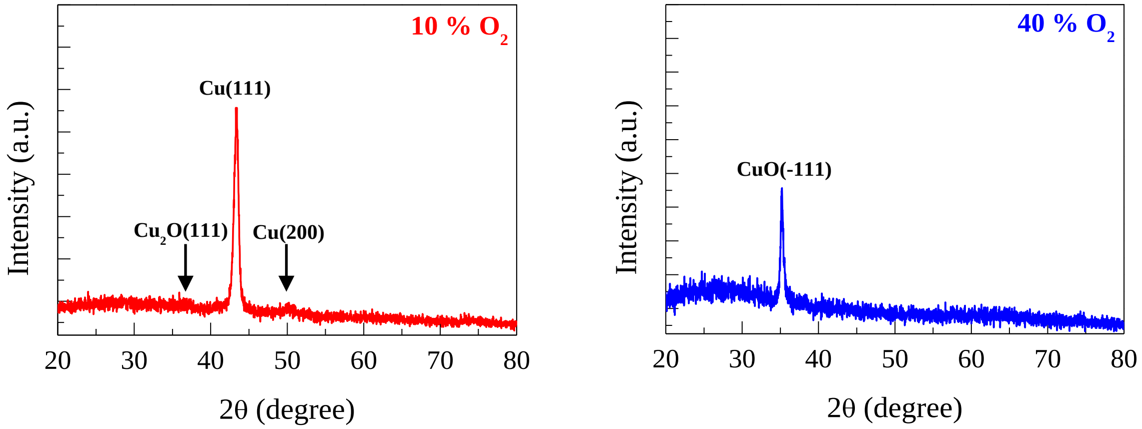


**Fig. S1** X-ray diffraction patterns of the copper oxide electrode films deposited with 10% and 40% oxygen flow rates.

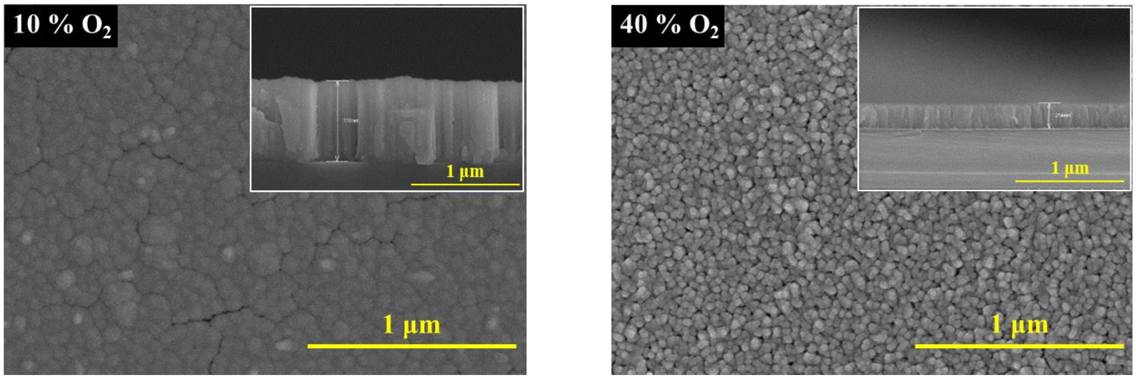


**Fig. S2** Plane and cross-sectional (inset) SEM images of copper oxide electrode films deposited with 10% and 40% oxygen flow rates.

**Table 1:** Compositional analysis of the copper oxide electrode films ($Cu_xO$) deposited with different oxygen flow rate percentages.

| Film | % of oxygen flow rate during deposition | At. % of Cu | At. % of O | *x* |
|---|---|---|---|---|
| $Cu_xO$ | 10 | 86.33 | 13.67 | 6.32 |
| | 20 | 70.21 | 29.79 | 2.36 |
| | 30 | 53.14 | 46.86 | 1.13 |
| | 40 | 51.68 | 48.32 | 1.07 |
